# Lytic and Chemotactic Features of the Plaque-Forming Bacterium KD531 on *Phaeodactylum tricornutum*

**DOI:** 10.3389/fmicb.2017.02581

**Published:** 2017-12-21

**Authors:** Zhangran Chen, Wei Zheng, Luxi Yang, Lisa A. Boughner, Yun Tian, Tianling Zheng, Hong Xu

**Affiliations:** ^1^Key Laboratory of the Ministry of Education for Coastal and Wetland Ecosystem, School of Life Sciences, and State Key Laboratory of Marine Environmental Science, Xiamen University, Xiamen, China; ^2^State Key Laboratory of Cellular Stress Biology, School of Life Sciences, Xiamen University, Xiamen, China; ^3^Center for Microbial Ecology, Michigan State University, East Lansing, MI, United States

**Keywords:** *Phaeodactylum tricornutum*, *Labrenzia* sp. KD531, chemotaxis, algal biomass, algal plaque

## Abstract

*Phaeodactylum tricornutum* is a dominant bloom forming species and potential biofuel feedstock. To control *P. tricornutum* bloom or to release lipids from *P. tricornutum*, we previously screened and identified the lytic bacterium *Labrenzia* sp. KD531 toward *P. tricornutum*. In the present study, we evaluated the lytic activity of *Labrenzia* sp. KD531 on microalgae and investigated its lytic mechanism. The results indicated that the lytic activity of KD531 was temperature- and pH-dependent, but light-independent. In addition to *P. tricornutum*, KD531 also showed lytic activity against other algal species, especially green algae. A quantitative analysis of algal cellular protein, carbohydrate and lipid content together with measurements of dry weight after exposure to bacteria-infected algal lysate indicated that the bacterium KD531 influenced the algal biomass by disrupting the algal cells. Both chemotactic analysis and microscopic observations of subsamples from different regions of formed plaques showed that KD531 could move toward and then directly contact algal cells. Direct contact between *P. tricornutum* and KD531 cells was essential for the lytic process.

## Introduction

Increasing evidence has shown that algal blooms now occur in many regions all over the world (Glibert and Burford, 2017; Gobler et al., 2017). Consequently, controlling and eliminating algal blooms have become a significant issues in environmental science (Le et al., 2010). Many methods are currently being tested for controlling algal blooms, including physical, chemical and biological techniques (Anderson, 2009). However, the use of algicidal bacteria to lyse algal cells has been shown to be more advantageous than chemical or physical methods (Prajapati et al., 2013).

Algicidal bacteria kill algae via two approaches. Indirect attack is the first mechanism by which algicidal bacteria produce algicidal substances without directly contacting algal cells; these algicidal substances then have an algicidal effect on algal growth. The second approach involves direct attack, where algicidal bacteria directly contact with the algal cells and then lyse them (Li et al., 2014b). There have been numerous reports on the indirect algicidal mechanisms adopted by algicidal bacteria. Guan et al. (2015) reported that haptophyte *Phaeocystis globosa* cells treated with the algicidal bacterium *Bacillus* sp. LP-10 died primarily due to photoinhibition induced by the bacterial infection. Cai et al. (2016) extracted an algicide from *Streptomyces alboflavus* RPS that could completely lyse *P. globosa* cells within 2 days. Zhang et al. (2013) reported that the algicidal activity of *Streptomyces alboflavus* RPS was sensitive to temperatures at and above 50°C, but insensitive to pH values from 3 to 11, while Li et al. (2014a) reported that algicidal material released by *Mangrovimonas yunxiaonensis* strain LY01 retained high algicidal activity even at 121°C. However, less research has been performed to investigate direct algicidal features and mechanisms than those of indirect algicidal modes. Furusawa et al. (2003) reported that *Saprospira* sp. SS98-5 lysed the cells of the diatom *Chaetoceros ceratosporum* via a direct algicidal method, as evidenced by partially degraded diatom cell walls at sites contacted by these bacteria. Li et al. (2016) reported that *Chitinimonas prasina* LY03 has direct algicidal activity against *Thalassiosira pseudonana* through the production of chitinase, which degrades the algal cell walls after direct contact between algal and bacterial cells. As has been reported, *Saprospira* spp. are general predators of bacteria and algae (Sangkhobol and Skerman, 1981) and must attach to their prey. Most algicidal *Cytophaga* also require attachment, although there are some exceptions. Their attachment to algae and ability to degrade cell surface macromolecules make *Cytophaga* well-suited for an algicidal lifestyle (Mayali and Azam, 2004).

Despite these findings, research on the direct algicidal mechanisms of bacteria on marine microalgae has rarely been considered. The marine microalgae *Phaeodactylum tricornutum* is a dominant bloom forming species that spreads widely in marine environments. In addition, 30–45% of the total lipids produced by *P. tricornutum* are polyunsaturated fatty acids (PUFAs) (Fajardo et al., 2007), of which eicosapentaenoic acid (EPA) is the major constituent, making *P. tricornutum* a potential biofuel feedstock. Algal-lysing bacteria are important for the control of *P. tricornutum* blooms or for the release of PUFAs from *P. tricornutum*. In our previous research, we found that *Labrenzia* sp. KD531 within *Rhodobacteraceae* showed direct algicidal activity against *P. tricornutum. Rhodobacteraceae* has been reported to have algicidal activity (Kim et al., 2008; Zheng et al., 2015), by taking advantage of the organic matter released by phytoplankton during an *Akashiwo sanguine* blooms (Yang et al., 2015). In view of its direct algicidal features and the potential applications in lysing biofuel microalgae, here, we investigated the relationship between the *Labrenzia* sp. KD531 and *P. tricornutum*.

## Materials and Methods

### Algal Cultures and Lytic Bacteria

Cultures of the experimental axenic alga *P. tricornutum* MEL106, were supplied by the State Key Laboratory of Marine Environmental Science (Xiamen University). Cultures of *P. tricornutum* were incubated in sterile f/2 medium (Guillard, 1975) that was prepared with 0.45 μm (Millipore, Bedford, MA, United States) filtered seawater at 20 ± 1°C under a 12/12-h light-dark cycle of approximately 50 μmol photons m^-2^s^-1^.

A total of 23 other exponentially growing algal species besides *P. tricornutum* MEL106 were included to examine the host specificity of bacteria-infected algal lysate (BIAL), which was tested by adding fresh BIAL to 10% (vol/vol), followed by culturing under the conditions mentioned above at 20°C for 14 days. These algal species were: *Platymonas subcordiformis, Chlorella vulgaris*, *Platymonas helgolandica, Chlorella, Prasinophyceae, Phaeocystis globosa, Dicrateria inornata, Isochrysis galbana, Heterosigma akashiwo, Chattonella marina, Alexandrium catenella* DH01, *Alexandrium tamarense* DH01, *Alexandrium minutum* TW01, *Scrippsiella trochoidea* XM01, *Prorocentrum donghaiense, Chaetoceros compressus, Thalassiosira pseudonana, Amphiprora alata, Thalassiosira weissflogii, Asterionella japonica* and *Skeletonema tropicum* which were provided by State Key Laboratory of Marine Environmental Science at Xiamen University, and *Microcystis aeruginosa* and *Dunaliella salina*, which were provided by Professor Yahui Gao at Xiamen University.

*Labrenzia* sp. KD531 that was grown in PTF medium formed plaques on *P. tricornutum* agar plates (Chen et al., 2015), and agar blocks within the single, newly plaques formed by *Labrenzia* sp. KD531 (MCCC 1K00274) were excavated, soaked in f/2 media overnight and then added to a large culture volume of exponentially growing *P. tricornutum* culture (approximately 2 × 10^6^ cells mL^-1^) for 20 days to generate stable BIAL. Fresh BIAL was prepared by adding 10% of the last BIAL to fresh exponentially growing *P. tricornutum*.

### Stability of the Algicidal Activity of the BIAL

Fresh BIALs were incubated at 4, 20, 30, 40, 50, 60, and 121°C for 1 h and then added to late exponential phase or stationary-phase *P. tricornutum* cultures (approximately 3 × 10^7^ cells mL^-1^) to determine the effect of temperature on its algicidal activity. To investigate whether light could affect the algicidal activity of the bacterium, fresh BIAL and *P. tricornutum* were mixed and incubated (20°C) in either the light or dark for 7 days. Moreover, to detect the effects of pH on the stability of the BIAL, the BIAL pH was adjusted to 6 and 7 using 0.1 M HCl and to pH values from 8 to 10 with 0.1 M NaOH. The pH-adjusted BIALs were then stored at room temperature for 2 h, after which the BIALs were re-adjusted back to their initial pH value, with an unaltered BIAL serving as a control. The pH-adjusted BIAL samples (10% v/v) were subsequently inoculated into the *P. tricornutum* cultures for 10 days. Additionally, to evaluate the effect of culturing temperature on algicidal activity, fresh BIAL was added to *P. tricornutum* cultures and then placed at 4, 20, 28, or 37°C for 10 days. We measured the chlorophyll a (Chl a) content per unit volume of culture and quantified the algicidal activity by evaluating decrease in Chl a content in infected cultures relative to that of an uninfected culture. Briefly, all algal cultures (20 mL) including both infected and uninfected cultures were pelleted using centrifugation at 5,000 × *g* for 10 min, precipitated with 5 ml of 90% ethanol to obtain a suspension, thoroughly vortexed and then incubated at -20°C overnight to extract chlorophyll. Thereafter, the suspensions were centrifuged at 8,000 × *g* for 10 min to remove cell debris. Absorbance value at wavelengths of 664, 645, and 630 nm were determined (ELO71139140, Varian Australia Pty Ltd., Australia), and the Chl a concentration was calculated using the formula:

$$Chl\,\;a\left({mgL}^{-1}\right)=11.64A_{664}-2.16A_{645}-0.10A_{630}\,\;(Kim\,\;et\,\;al.,\;2009).$$

The algicidal rate was calculated using the formula:

$$\text{Algicidalrate}\left(\%\right)=(Chl\,\;a_{Con}-Chl\,\;a_{Tre})/Chl\,\;a_{Con}\times 100\%,$$

Chl a_Con_ and Chl a_Tre_ denote the Chl a concentrations of the control and treatment, respectively.

### Microscopic Observations

For the scanning electron microscope (SEM) (JSM-6390, JEOL Co., Japan) observations, agar blocks from within, on the boundary and outside of the plaques formed on the algal plates were excavated and soaked in f/2 liquid medium for 1 h. The agar blocks were then fixed in sodium phosphate buffer (PBS, 50 mM, pH 7.4) containing 2.5% glutaraldehyde (v/v) for 2 h and then gently rinsed twice with PBS, followed by post-fixation in 1% OsO_4_ in the same buffer for 2 h. The samples were then gently rinsed twice with PBS, followed by dehydration in a gradient ethanol series (30, 50, 70, 90, 95, and 100%) and were finally incubated in pure tertiary butanol at 4°C overnight. The samples were critical point dried and mounted on stubs, and the preparations were then sputter-coated with gold-palladium at 60:40 and 25:30 nm. For transmission electron microscopy (TEM) (JEM-2100HC, JEOL Co., Japan), agar blocks were prepared from within, on the boundary and outside of the plaque zones formed on the algal plates and were then suspended in f/2 liquid medium for 15 min and processed as previously described (Chen et al., 2014).

### Effects of the BIAL on *P. tricornutum* Biomass

Total cellular protein, carbohydrate and lipid contents were independently determined, and the cellular dry weight was measured. Algal cells were pelleted using centrifugation at 4,000 × *g* for 5 min, followed by rinsing in 1 mL of PBS (50 mM, pH 7.8). To determine the cellular protein content, the proteins were extracted according to a reported method (Zhang et al., 2013) and detected using a “Coomassie brilliant blue protein analysis kit” (Nanjing Jiancheng Bioengineering Institute, China) with bovine serum albumin as the standard. The carbohydrate content was determined using the phenol-sulfuric acid colorimetric method, with glucose as the standard (Li et al., 2014b). The algal cells were concentrated at 4,500 × *g* for 10 min, vacuum dried and measured to determine the total dry weight, and the total lipids were then extracted using a mixture of chloroform and methanol (Lee et al., 2010).

### Chemotactic Effect of the BIAL on *P. tricornutum* Nutrition

To prepare a soft agar medium, 100 mL of MM_2_ culture medium (Sohn et al., 2004) was mixed with agar to a final concentration of 0.18% (w/v), and autoclaved at 121°C for 21 min. After autoclaving, when the temperature reduced to 50°C, 10 mL of BIAL was quickly added to the medium, evenly mixed and poured into plates. A total of 100 mL of exponentially growing *P. tricornutum* (approximately 2 × 10^6^ cells mL^-1^) was centrifuged at 20°C for 10 min at 5,000 × *g*, and the supernatant and pelleted cells were independently transferred to sterile tubes. For different treatments of algae, a portion of the collected algae cells were autoclaved at 121°C for 21 min, and the remaining pelleted algal cells were lysed using an Ultrasonic Cell Disruption System (NingBo Scientiz Biotechnological Co., Ltd., China) (120 W, 5 s:5 s, 80 times). Subsequently, 10 mL of the centrifuged algal culture supernatant and sonicated cell pellet were individually and gently applied to the center of the BIAL/agar plates described above, with H_2_O serving as a control. All the plates were kept in the dark at 20°C for 2 days, which was sufficient for the observation of KD531 chemotaxis toward the central nutrition zone (i.e., portions of the algal culture).

### Statistical Analysis

All data are presented as the mean ± standard error of the mean and were evaluated using one-way analysis of variance with three biological replicates, followed by the least significant difference test, with *p* < 0.01 and *p* < 0.05.

## Results

### Sensitivity of the BIAL to Temperature and the Effect of Incubation Temperature on the Algicidal Activity of the BIAL on *P. tricornutum*

**FIGURE 1:**
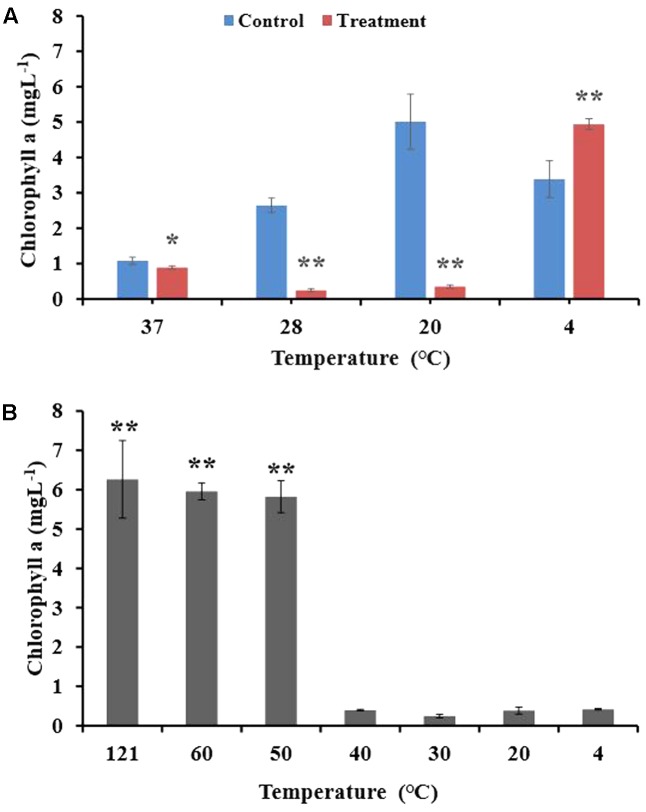
Algicidal stability of the BIAL at different culturing temperatures and its temperature sensitivity during storage. **(A)** Effect of culturing temperature on the algicidal activity of the BIAL. **(B)** Temperature sensitivity of the BIAL, Chl a under 20°C served as control. ^∗^*p* < 0.05 and ^∗∗^*p* < 0.01 represent significant differences compared to the control cells.

After incubating the algal culture and BIAL mixture for 7 days at different temperatures (4, 20, 28, and 37°C), the Chl a content was measured to determine the temperature that retained the highest KD531 algicidal activity. As seen in **Figure 1A**, *P. tricornutum* grew best at 20°C, which is the typical culturing temperature for this species. At the other tested temperatures, the algal growth rate was either reduced or inhibited. The algicidal activity of KD531 at 4°C was not sufficient to lyse the algae, as the Chl a concentration of *P. tricornutum* remained high. However, at higher temperatures (20°C and 28°C), the algicidal activity remained significant, even though the growth of *P. tricornutum* was reduced. To understand how temperature affects the algicidal activity of KD531, the BIAL was exposed to a variety of temperatures before algal culture inoculation. **Figure 1B** shows that algal cultures with BIAL additions that were exposed to temperatures higher than 50°C had Chl a concentrations of approximately 6 mgL^-1^, while significantly lower residual Chl a content was observed below 40°C, indicating that the BIAL lost its algicidal activity at temperatures above 50°C. Based on these results, the algicidal activity of BIAL was indicated to be temperature-dependent.

### Effect of Light and pH on the Lytic Capability of the BIAL

**FIGURE 2:**
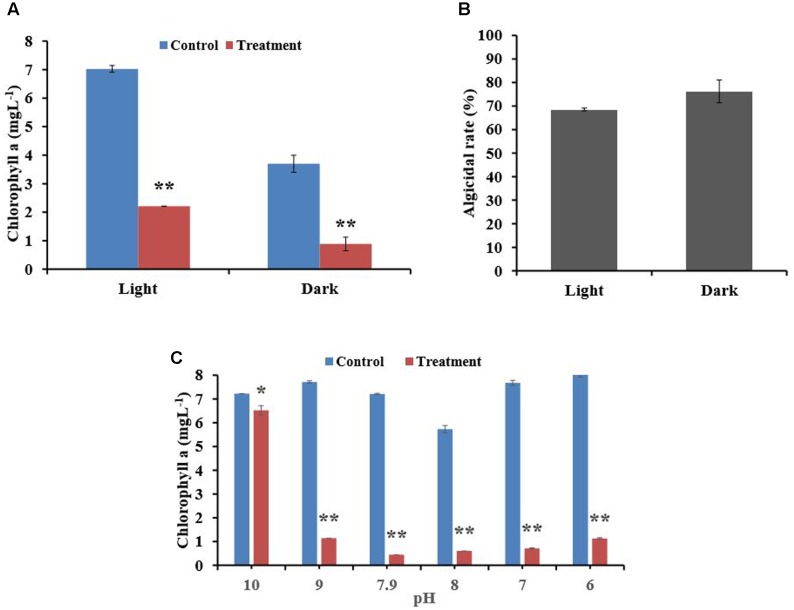
Effects of light and pH on the algicidal activity of the BIAL. **(A)** The algal Chl a content after incubation with the BIAL under light and dark conditions. **(B)** The algicidal rate of the BIAL under light and dark conditions. **(C)** The algicidal stability of the BIAL at different pH values. ^∗^*p* < 0.05 and ^∗∗^*p* < 0.01 represent significant differences compared to the control cells.

To understand the effect of light on algicidal activity, we incubated mixtures of algal culture and BIAL for 7 days in either continuous light or darkness. As seen in **Figure 2**, light was necessary for algal growth because *P. tricornutum* growth was inhibited in the dark. The algicidal activity of the BIAL was not affected by light; the algicidal rate was 75% under dark conditions and nearly 70% under light (**Figure 2B**). Hence, light is not an essential factor for KD531 algicidal activity (*p* > 0.05). To understand how pH affected the algicidal activity of KD531, the pH of the algal culture and BIAL mixture was adjusted and incubated similar to that of the light condition for 7 days. **Figure 2C** shows that *P. tricornutum* can adapt to a relatively wide pH range (from pH 6–10); therefore, it was not surprising that the pH does not significantly affect the algicidal activity of the BIAL on *P. tricornutum*. Moreover, the algicidal activity of the BIAL remained stable between pH 6 and 9, during which the Chl a measurements were approximately 1 mgL^-1^. Nevertheless, KD531 lost its algicidal activity when the pH reached 10, indicating that the algicidal activity of the BIAL was sensitive to highly alkaline pH conditions.

### Effect of the BIAL on the Growth of Other Algal Species

Among the algal species within Bacillariophyta, the BIAL showed lytic activity only against the host algae *P. tricornutum* (**Table 1**). The BIAL had no lytic activity on algae from Pyrrophyta, Chrysophyta and Xanthophyta. Interestingly, the BIAL could form plaques on *Dunaliella salina, Platymonas subcordiformis, Chlorella vulgaris, Platymonas helgolandica, Chlorella* sp., and *Prasinophyceae* sp. agar plates, strongly suggesting that KD531 is a lytic bacterium that primarily targets Chlorophya. Nonetheless, with lytic activity toward *Microcystis aeruginosa* and *P. tricornutum*, the BIAL showed algicidal activity against a spectrum of algal taxa.

**Table 1:** Lytic capabilities of KD531 on algal species.

Division	Algal species	Lytic ability
Chlorophyta	*Dunaliella salina*	+
	*Platymonas subcordiformis*	+
	*Chlorella vulgaris*	+
	*Platymonas helgolandica*	+
	*Chlorella* sp.	+
	*Prasinophyceae* sp.	+
Chrysophyta	*Phaeocystis globosa*	-
	*Dicrateria inornata*	-
	*Isochrysis galbana*	-
Cyanophyta	*Microcystis aeruginosa*	+
Xanthophyta	*Heterosigma akashiwo*	-
	*Chattonella marina*	-
Pyrrophyta	*Alexandrium catenella* DH01	-
	*Alexandrium tamarense* DH01	-
	*Alexandrium minutum* TW01	-
	*Scrippsiella trochoidea* XM01	-
	*Prorocentrum donghaiense*	-
Bacillariophyta	*Phaeodactylum tricornutum*	+
	*Chaetoceros compressus*	-
	*Thalassiosira pseudonana*	-
	*Pmphiprora alata*	-
	*Thalassiosira weissflogii*	-
	*Asterionella japonica*	-
	*Skeletonema tropicum*	-


*“+” denotes lytic ability while “-” indicates no lytic ability.*

### SEM and TEM Observations of Different Plaque Regions

**FIGURE 3:**
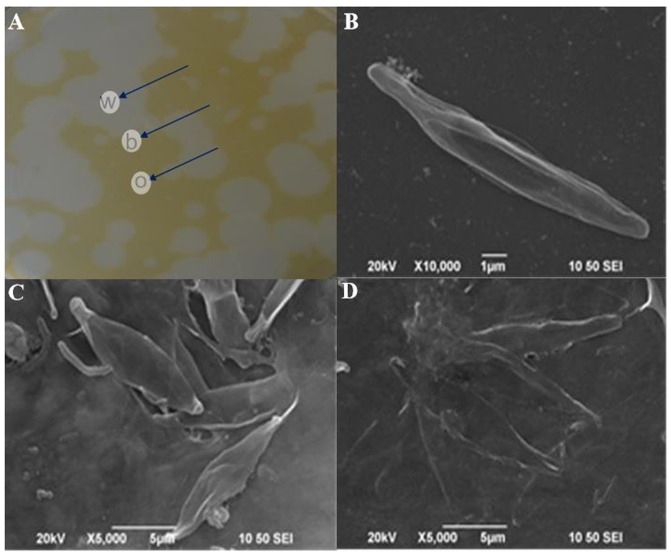
Scanning electron microscope (SEM) images of the morphological changes of *P. tricornutum* cells from within, on the boundary and outside of the plaques. **(A)** Different plaques zones on an algal agar plate; **(B)** SEM images of outer plaque regions; **(C)** SEM images from the plaque boundaries; **(D)** SEM images from within the plaques. w, within; b, boundary; o, outer plaque regions. Scale bars: **(B)** 1 μm, **(C)** and **(D)** 5 μm.

SEM analysis revealed the typical morphological changes in *P. tricornutum* cells from various regions of plaques including those within (w), on the boundary (b) and outside (o) of the plaques (**Figure 3**). **Figure 3A** showed the plaques formed by KD531 after 10 days; we excavated agar blocks from different regions for direct observation. In the outer region o (**Figure 3B**), the *P. tricornutum* cells were intact, and there were no algicidal bacteria interacting with the algae. However, in the agar block from the plaque boundary (**Figure 3C**), more bacteria were adhered to algal cells, and some of the algal cells were lysed. In the central regions of the plaques, most of the algal cells were lysed (**Figure 3D**).

**FIGURE 4:**
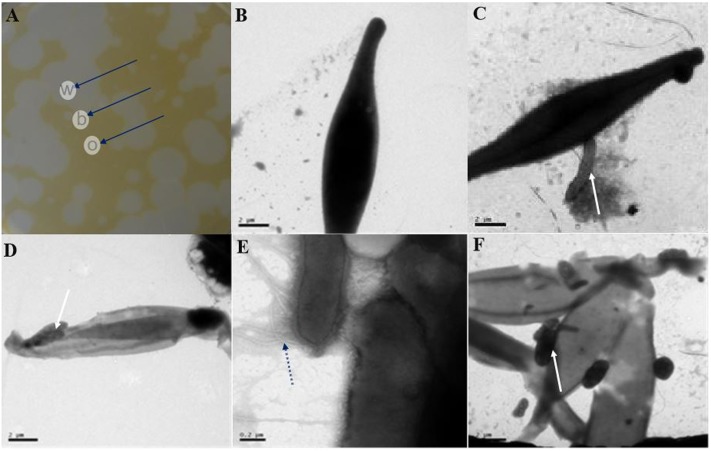
Transmission electron microscopy (TEM) images of the morphological changes of *P. tricornutum* cells from within, on the boundary and outside of the plaques. **(A)** Different plaques zone on an algal agar plate. **(B)** TEM images of outer plaque regions. **(C,D)** TEM images from the plaque boundaries. **(E,F)** TEM images from within the plaques. w, within; b, boundary; o, outer plaque regions. Scale bars: **(B–D,F)** 2 μm and **(E)** 0.2 μm. Black arrows in **(A)** indicate the different parts of the plaques, and white arrows in **(C,D,F)** indicate the site where the bacterial cells contacted the algal cells, while the black dotted arrow in **(E)** indicates the microtubule structure of a bacterium.

To study the relationship between the algal and lytic bacterial cells, we used TEM to observe the aforementioned agar block plaque samples (**Figure 4A**). Again, the algal cells remained intact in the o regions (**Figure 4B**), indicating that the lytic bacteria were not affecting the algal cells. However, in the b (**Figures 4C,D**) and w (**Figures 4E,F**) areas, more than one bacteria cell was directly interacting with host cells. Increasing degrees of algal cells degradation were observed from the outermost to the innermost plaque zones, which is understandable because as the diameter of the plaques extended outward, the bacterial lytic activity caused new algal cells to lyse. Microtubule-like structures around the bacterium (**Figure 4E**) could be clearly observed at higher magnifications, consistent with the previously published results (Van Etten et al., 1983).

### Influence of the BIAL on *P. tricornutum* Biomass

**FIGURE 5:**
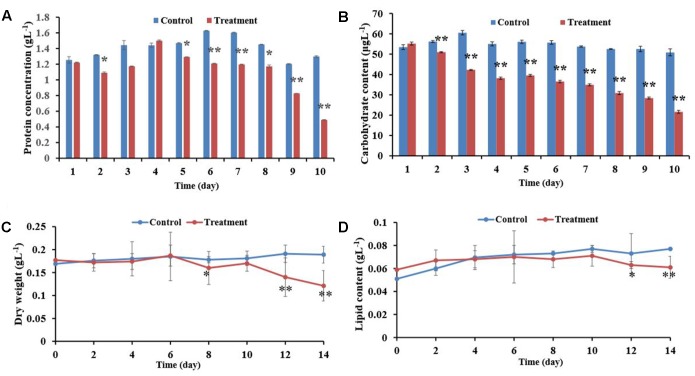
Effects of the BIAL on *P. tricornutum* biomass. **(A)** Algal cellular protein content after exposure to the BIAL for 10 days. **(B)** Carbohydrate content after exposure to the BIAL for 10 days. **(C)** Dry weight of the algal cells after lysis by the BIAL for 14 days. **(D)** Assay of the algal lipid content of cells lysed by the BIAL over 14 days. ^∗^*p* < 0.05 and ^∗∗^*p* < 0.01 represent significant differences compared to the control cells.

**Figure 5** showed the changes in algal cell biomass (cellular protein, carbohydrate and lipid content and cellular dry weight) that were observed after *P. tricornutum* cultures were exposed to the BIAL. In the first 5 days, the presence of the lysate did not cause significant changes, indicating that in the early stages of infection, the BIAL had a minimal effect on the algal cellular protein content. The *P. tricornutum* cellular protein content remained relatively constant (approximately 1.2 gL^-1^) until day 8, after which the protein content decreased drastically. Nevertheless, within 10 days of BIAL exposure, the *P. tricornutum* protein content was 38% of that of the control (**Figure 5A**).

Alternatively, the *P. tricornutum* cellular carbohydrate contents decreased significantly after only 2 days of exposure to the BIAL (**Figure 5B**), and after 10 days, the carbohydrate content was approximately 40% (*p* < 0.01) of that of the no-BIAL control. Although the carbohydrate contents decreased in proportional to the duration of BIAL exposure, reducing by approximately 35 μgL^-1^ from day 4 to day 7, the carbohydrate content decreased continuously to approximately 20 μgL^-1^ on day 10 (43% of that of the no-BIAL control).

When *P. tricornutum* was exposed to the BIAL, the algal cell dry weight remained relatively constant at 0.18 gL^-1^ until day 6 when it gradually decreased to 0.12 gL^-1^ (**Figure 5C**). The total lipid content of the algal cells persisted at approximately 0.075 gL^-1^ in the no-BIAL control, decreasing to 0.06 gL^-1^ by the tenth day (**Figure 5D**). However, the significant difference could only be recognized until day 6, after which it gradually decreased (*p* < 0.05).

### Chemotactic Capabilities of KD531

**FIGURE 6:**
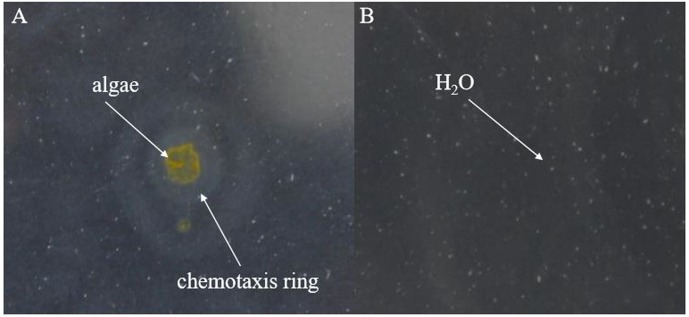
Formation of chemotactic rings by the lytic bacterium KD531. **(A)** Ten microliters of a *P. tricornutum* cultures was spotted on a KD531-containing soft agar plate (0.18% agar content), and a chemotactic ring formed. **(B)** H_2_O was spotted on the same medium, serving as a control.

After preparing the BIAL in soft agar plates, the plate center was spotted with drops (10 μL) from *P. tricornutum* cultures and incubated in the dark for 2 days, after which a chemotactic ring formed. As shown in **Figure 6B**, no chemotactic rings formed on the control H_2_O spotted plates, excluding the possibility that a ring formed as a result of diffusion (of H_2_O or other liquids).

**FIGURE 7:**
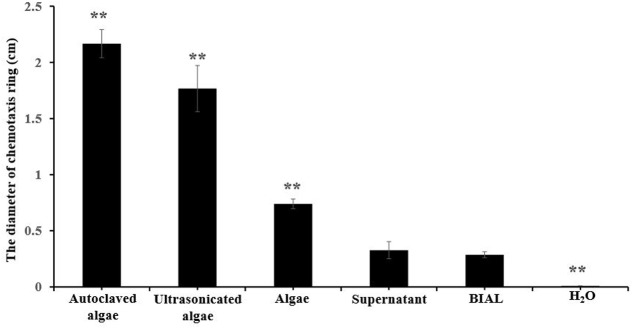
Chemotactic rings formed by different *P. tricornutum* culture treatments. Autoclaved algae, a portion of the algal cells were treated at 121°C; ultrasonicated algae, a portion of the algal cells subjected to ultrasonic cell disruption; algae, an algal cell pellet obtained from centrifugation; supernatant, culture liquid obtained after centrifugation; BIAL, new BIAL produced by adding BIAL to *P. tricornutum* cultures; H_2_O, the negative control. All error bars indicate the SE of the three biological replicates. Significant difference were based on the pair comparison between BIAL and the other treatment. ^∗^*p* < 0.05 and ^∗∗^*p* < 0.01 represent significant differences compared to the control cells.

Based on the results shown in **Figure 6**, we speculated that either a chemotactic signal was released from *P. tricornutum* cells, or that the cellular surface itself might induce the formation of a chemotactic ring, which did not appear at all on the control H_2_O spotted plates. The maximum ring diameter (2 cm) appeared when autoclaved algae were spotted on the plates, and the second-largest diameter (1.8 cm) occurred with ultrasonicated algae group (**Figure 7**), indicating that the disruption of algal cells by drastic methods helps release chemical materials that induce chemotaxis. After culture centrifugation, both the supernatant and the algae pellets resulted in the formation of rings with diameters of 0.3 and 0.8 cm, respectively. Although most of the algae cells were disrupted by the BIAL, some nutritional compounds remained in the algal cell fragments, which may have enabled the formation of a ring 0.2-cm in diameter.

## Discussion and Conclusion

Due to harmful algal blooms (HABs) regulation and biofuel-producing microalgae utilization, algicidal bacterial have attracted worldwide attention as a potentially effective means to control bloom development and to lyse microalgal cells for downstream biofuel production (Qianlong et al., 2016; Zhou et al., 2016). Algicidal bacteria kill phytoplankton through two different approaches, i.e., direct attack and the production of lethal substances. Few studies concerning bacterial algicidal ability and the relationship between bacteria and algae have been performed, especially in terms of direct algicidal activity. Direct attack requires the presence of bacterial cells. Imai et al. (1993) reported that *Cytophaga* sp. J18/M01 killed five raphidophycean flagellates, four diatoms, and one dinoflagellate via direct attack. They also determined that the algicidal activities of strains K and D depend on their prey phytoplankton species and not extracellular products (Imai et al., 1995). Furusawa et al. reported that *Saprospira* sp. SS98-5 could lyse the cells of the diatom *Chaetoceros ceratosporum* by direct contact between algal and bacterial cells (Furusawa et al., 2003). Li et al. (2016) reported that *C. prasina* LY03 directly kills the diatom *T. pseudonana* by producing a chitinase, which lyses the algal cell walls. In addition, bacterial plaque formation on algae is considered to be a visual phenomenon that indicates direct algicidal activity (Van Etten et al., 1983; Sakata et al., 1991). Our previous research showed that *Labrenzia* sp. KD531 could form plaques when grown on *P. tricornutum*, which were proven to be caused by direct attack since the 0.22-μm cell-free filtrate had no lytic activity (Chen et al., 2015). Moreover, in this study, both the TEM and SEM observations revealed that direct contact between the algal and bacterial cells was essential. Currently, few studies regarding the algicidal activity of the genus *Labrenzia* against diatoms have been reported, and in depth analysis of direct algicidal mechanisms are lacking. Interestingly, we noticed that the BIAL here had the ability to lyse not only *P. tricornutum* but also green algae (Chlorophyta). Imai et al. (1993) also reported that the algicidal bacteria *Alteromonas* sp. strains S and R and *Cytophaga* sp. J18/M01, which directly attack their prey, had no effective algicidal activity against the armored (or thecate) dinoflagellates, *Alexandrium tamarense* and *Heterocapsa circularisquama*, but they had lytic activity on algae within Chlorophyta (Imai et al., 1993). Our study agrees with speculation in this report that the algicidal activity depends on the morphological and ecophysiological characteristics of the host algae. However, the underlying reason for the specific algicidal ability of the BIAL against green algae is worthy of further investigation.

Bacteria from the genus *Labrenzia* grow optimally at temperatures of 28–30°C (Biebl et al., 2007), which explains the high algicidal activity of KD531 observed near this temperature range. KD531 could not grow at higher temperatures, and its algicidal activity was lost after incubation at the higher temperatures; therefore, the activity of the BIAL was sensitive to temperatures of 50°C and above. Most research regarding the influence of temperature on algicidal activity has focused on indirect algicidal attack; few studies have reported data related to direct algicidal attacks. The optimum temperature for *Pseudoalteromonas* sp. strain A28 to release a protease capable of killing *Skeletonema costatum* cells was found to be 30°C (Lee et al., 2000), which is an example of indirect algicidal attack. Direct algicidal attack involves one-to-one contact between algal and bacterial cells. As previously reported, temperature may affect the ability of a bacterium to swim (Gerber et al., 1973), but almost no literature regarding direct algicidal mechanisms has been reported. We speculate that high temperature may influence the movement of the bacterium KD531. *Labrenzia* sp.KD531 had stable lytic activity toward *P. tricornutum* at pH values of 6–9, while its lytic activity lost when the pH reached 10; these results are in accordance with a report that bacteria within the genus *Labrenzia* grow at pH values of 6.0–9.0 (Biebl et al., 2007).

In the co-culture system, the BIAL had an adverse effect on the growth of the host algae, as both its carbohydrate and lipid content decreased gradually over 10 days of incubation. A significant discrepancy between the dry weight cell biomass and Chl a content in the culture medium of infected cells relative to that of the control was observed; we speculated that different quantification methods were responsible for this finding. Chl a content was quantified using a spectrophotometer, and it reflects active, intact algal cells, not damaged cells. Conversely, the cell dry weight was quantified by weighing centrifuged, dried pellets, which included intact cells and debris from dead cells. Although the bacterial fractions could not be easily removed, the contaminated bacteria exerted only a limited influence on the carbohydrate, protein and lipid contents of the algal cultures. In addition, the BIAL demonstrated algicidal activity against microalgae other than *P. tricornutum*, making this organism a promising candidate for disrupting microalgae during the production of biofuels. Future research is needed to determine the influence of the BIAL on other microalga. Of course, undesired microbial cross-contamination during large scale biomass cultivation is still a practical concern. As demonstrated herein, the bacterium KD531 could display algicidal activity against microalga mostly within the Chlorophyta; the verification of the algicidal activity of KD531 is imperative before utilizing this bacterium for large volume algal biomass cultivation.

Chemotaxis is a behavioral response exhibited by many bacteria in which they have the ability to move toward or away from nutrients and/or chemicals in the environment (Parales and Harwood, 2002). Our investigations of the ability of KD531 to move toward *P. tricornutum* indicates that chemotaxis occurred in our system. This phenomenon was even clearly observed around the source of the nutrition, but chemotactic rings were still observed in the absence of nutrients. As the algicidal bacteria in the BIAL were uniformly mixed with soft agar, the bacteria must be evenly distributed throughout the plate. A decreasing nutritional concentration gradient would expand outward from the centrally spotted nutritional additive, and bacteria farther from the nutritional center would move inward if able, leading to a growing chemotactic ring. Hence, we speculated that the algicidal *Labrenzia* sp. KD531 bacteria showed chemotactic abilities when they sensed the chemotactic signal from *P. tricornutum*. In addition, we tested both autoclaved and ultrasonicated *P. tricornutum*; the former was superior to the latter in terms of lysis efficiency and sufficient nutrition, and it more strongly induced chemotaxis. Moreover, the chemotactic signal is likely heat-resistant as it still attracted *Labrenzia* sp. KD531 after autoclaving. Based on our results regarding the formed plaques and chemotactic abilities, we speculate that *Labrenzia* sp. KD531 responds to a signal released by *P. tricornutum*, and upon interacting with the algal cells, the algicidal activity of the bacterium is initiated, leading to plaque formation.

Although this research revealed the algicidal features and chemotactic abilities of *Labrenzia* sp.KD531, further investigations will be needed to reveal more details about its potential algicidal mechanism, which could involve changing algal cell morphology, or physiology, at the biochemical or genetic levels. In addition, determining what contributes to the temperature or pH-dependent features of the algicidal activity of KD531 as well as the mechanism by which KD531 exhibits lytic activity by directly interacting with *P. tricornutum* will be important. Furthermore, identifying the algal chemotactic signal and bacterial chemoreceptors is required. Finally, for the algicidal abilities of *Labrenzia* sp. KD531 to be applicable on a large scale (for biofuel production or HAB treatment), many industrial safe guards would need to be implemented to prevent the unwanted infection of algal biomass.

Overall, the lytic activity of *Labrenzia* sp. KD531 remained stable up to 40°C in a pH range of 6–9, and light had almost no effect on its algicidal activity. In addition to *P. tricornutum*, the BIAL also showed stable lytic activity against other potential biofuel-producing microalgae within the Chlorophyta, such as *Chlorella vulgaris*. The algal biomass changes observed during the lytic process with *Labrenzia* sp. KD531 indicate that use of this organism might be an efficient method for the disruption of dense collections of algal cells to obtain beneficial materials for biofuel production. The formation of chemotactic rings and plaques indicated that certain chemicals within *P. tricornutum* might act as a stimulants to induce *Labrenzia* sp. KD531 to move toward and directly contact the algal cells, resulting in *P. tricornutum* cell disruption.

## Author Contributions

Conceived and designed the experiments: ZC, WZ, YT, and TZ. Performed the experiments: ZC, WZ, and LY. Analyzed the data: ZC, WZ, LY, LB, and TZ. Wrote the paper: ZC, TZ, and HX. Decided to publish: TZ and HX. All authors reviewed the manuscript.

## Conflict of Interest Statement

The authors declare that the research was conducted in the absence of any commercial or financial relationships that could be construed as a potential conflict of interest.
